# First diagnosis of septic arthritis in a dinosaur

**DOI:** 10.1098/rsos.160222

**Published:** 2016-08-03

**Authors:** Jennifer Anné, Brandon P. Hedrick, Jason P. Schein

**Affiliations:** 1 School of Earth, Atmospheric and Environmental Sciences, University of Manchester, Manchester M13 9PL, UK; 2 Department of Biology, University of Massachusetts Amherst, Amherst, MA 01003, USA; 3 Bureau of Natural History, New Jersey State Museum, Trenton, NJ 08625, USA

**Keywords:** palaeopathology, X-ray microtomography, hadrosaur

## Abstract

Identification and interpretation of pathologies in the fossil record allows for unique insights into the life histories of extinct organisms. However, the rarity of such finds limits not only the sample size for palaeopathologic studies, but also the types of analyses that may be performed. In this study, we present the first occurrence of a palaeopathology in a vertebrate from the Mesozoic of the East Coast of North America (Appalachia), a pathologic ulna and radius of an indeterminate hadrosaur from the Navesink Formation (New Jersey). X-ray microtomography allowed for both detailed and more accurate diagnosis of the pathologic condition as well as virtual conservation of the specimen. Based on extant archosaurian comparisons, the hadrosaur was diagnosed with severe septic arthritis affecting the proximal ulna and radius. Diagnosis was based on erosion of the joint and highly reactive periosteal bone growth and fusion of the elements. To the best of our knowledge, this is the first recorded account of septic arthritis in dinosaurs. The severity of the pathology suggests the animal suffered with this condition for some time before death. Unfortunately, only the ulna and radius were found. Thus, the extent to which the condition spread to other parts of the body is unknown.

## 1. Introduction

The Mesozoic terrestrial fauna of Eastern North America is poorly represented in the fossil record, consisting mostly of trackways and rare skeletal remains [1]. The Late Cretaceous exposures in particular produce extremely fragmentary terrestrial remains, which are often attributed to deposition by ‘bloat and float’ in the marine sediments [1,2]. Despite the condition of the fossils, dinosaurs from the Late Cretaceous Appalachia are evolutionarily significant in that they represent an entirely separate ecosystem from the Laramidian fauna; the western North America Late Cretaceous fauna well known for species such as *Tyrannosaurus* and *Triceratops* [1–3]. East Coast specimens are also of historic significance, with the Mesozoic exposures of New Jersey representing the birth place of academic vertebrate palaeontology in the Americas [1–3]. Unfortunately, many of the specimens are not only fragmentary, but are also infected by pyrite disease, which makes them extremely fragile. Given the rare state of such fossils, it was a surprise that a pathological specimen, which is rare even in well-sampled systems, was found in the Hungerford and Terry Inc. (Inversand) quarry in Gloucester County, NJ, USA [2].

Palaeopathology, the study of ancient diseases and trauma, offers a unique snapshot into the immunology and life histories of extinct animals [4–6]. These studies are most prominent in vertebrates because conditions that affect bone are much more likely to be preserved than soft tissue conditions. In addition, the most reliable method for palaeopathological diagnosis is histological analysis, which often requires destructive sampling via thin section preparation. This limits the number of species and specimens that can be sampled, due both to the rarity of the preserved condition and the desire to conserve specimens for future use through non-destructive analyses. The pathologic New Jersey hadrosaur (NJSM GP11961) represents a very rare find given the provenance and pathological condition. However, not only is the specimen rare, but it is also extremely fragile and in danger of ‘self-destruction’, which has been documented for many of the specimens collected from this site. This makes thin sectioning impossible, limiting the accuracy of potential diagnoses.

Fortunately, the twenty-first century has seen a large increase in both the power and availability of non-destructive analytical techniques such as X-ray microtomography (microCT, XMT). Such tools have allowed palaeopathologists to both increase the sample size and the integrity of their diagnoses through non-destructive internal analysis [5,7–10]. Here, we present XMT data from the pathological hadrosaur ulna and radius (NJSM GP11961) to (i) accurately diagnose the pathological condition, and (ii) digitally conserve a rare and important specimen.

## 2. Material and methods

### 2.1. Geology

NJSM GP11961 was collected from the Navesink Formation, approximately 1 m below the contact with the overlying Hornerstown Formation. The Navesink Formation is a massive, unconsolidated, medium- to coarse-grained glauconitic sand with minor amounts of terrigenous sandy clay. It is fossiliferous, extensively bioturbated and interpreted to have been deposited in a productive, shallow-water, inner shelf environment during a transgression [11]. A Late Maastrichtian age estimate for the unit has been supported by both stable strontium isotope analyses [12] and ammonite biostratigraphy [13]. The Navesink Formation preserves many invertebrate and vertebrate species common in the Late Cretaceous near shore marine sediments, such as bivalves, brachiopods, crustaceans, teleost fish, sharks, turtles and the occasional mosasaur and dinosaur.

### 2.2. Specimen

The specimen consists of a pathological ulna and radius from a hadrosaur (family Hadrosauridae) measuring 675 mm and 535 mm, respectively (preserved lengths). The specimen was assigned to Hadrosauridae through similarities with hadrosaurid material at Princeton University (D Parris 2016, personal communications). The ulna and radius were found fused, but have since been separated.

### 2.3. MicroCT

Both skeletal elements were scanned using a Nikon Metrology (X-Tek) HMXST225 MicroCT system at the Center for Nanoscale Systems at Harvard University. Each sample was embedded in styrofoam and mounted onto the X-Tek stage to ensure that the bones neither broke nor moved during scanning. Only the proximal ends (area of interest with pathology) were scanned, as full scans were not possible due to the size limitations of the X-Tek system. The entire bone may have been scanned using a medical CT system, but given the importance of high resolution for the diagnosis of the pathology, we deemed the use of the XMT scanner justified.

Before starting the scans, the system was autoconditioned with voltage set to 225 kV and current to 30 µA. After autoconditioning, samples were loaded and voltage and current were set to maximize contrast such that the greyscale values of the darkest pixels were at least 8000 along the thickest part of the samples. The specimens were then removed and the X-rays were switched off in order to eliminate any latent image from the detector. Samples were reloaded and the number of projections was set to ‘optimize’. The ‘frames per projection’ was left at the default setting of one. The source voltage and current settings were paired with 0.25 mm Cu filters to minimize beam hardening while maintaining the optimal spectral width and intensity (desired transmission through the specimen). The white target (brightest pixel on the scan) was set to 60 000 counts for the selected gain in order to reduce gain. In order to reduce scan time, ring artefacts were not minimized. Integration time was set at 1000 ms to achieve maximum imaging resolution. The radius was scanned at a voxel size of 96.82 µm with a voltage of 130 kV and current of 120 µA. The ulna was scanned at a voxel size of 127.03 µm with a voltage of 160 kV and current of 135 µA.

After scanning, the samples were imported into the proprietary CTPro3D software paired with the HMXST225 MicroCT to create a vgl file from the image stack. The vgl file was then imported into VGStudioMAX for reconstruction. The scans were modified for optimum contrast and then exported as jpg image stacks. These image stacks were then put into Materialize Mimics in order to reduce triangle count and delete floating objects. Materialize Mimics allows for visualization of slices in three views simultaneously (lateral, cranial and transverse), which can be used to assess microstructural changes in the bone qualitatively. In Mimics, files were exported into the associated Materialize 3-matic software in order to generate three-dimensional surface files. Image stacks, vgl and surface files are available to download through Dryad (http://dx.doi.org/10.5061/dryad.b84nh).

All results from the hadrosaur were then compared to known conditions in extant avians and reptiles for diagnosis of the pathological condition (table 1).

**Table 1.** List of possible conditions with descriptions and occurrences in extant reptiles and birds.

condition	description (skeletal)	extant occurrences
neoplasia	*osteoma*: small encapsulated nodules with disorganized bony trabeculae	avians, rare in wild individuals [14–18]
	*osteosarcoma*: growth around ends of long bones composed of sporadic trabeculae, islands of cartilage and fibrous connective tissue	crocodilians, extremely rare [19]
osteopetrosis	thickening of the diaphysis cortical wall in long bones	poultry [20]
osteomyelitis	a form of infection of bone or bone marrow [6]. Mostly degenerative in reptiles and birds with very little to no periosteal reactive bone growth [21]	avians [16–18,22–25]
		reptiles [21,26,27]
septic arthritis	infection of the synovium around joints, causing destruction of the articular cartilage, erosion of the joint and eventually ankylosing of bone [6]	crocodilians [19] sea turtle [28]
gout	lytic lesions around joints caused by urate crystals	reptiles, very common [4,21,27]
tuberculosis	‘punched-out’ lesions in the long bones	avian, very common [16,29]

## 3. Results

### 3.1. Ulna

**Figure 1.**
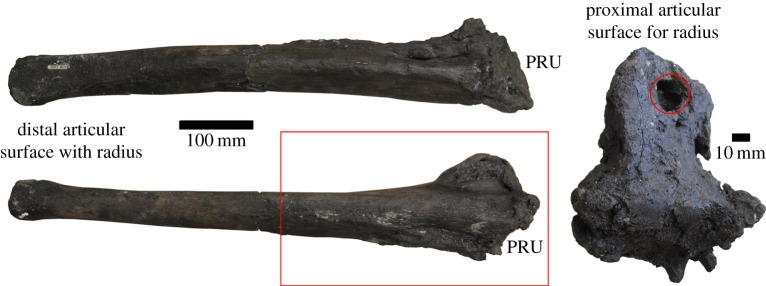
Pathological ulna from a hadrosaur (NJSM GP11961) with severe bone remodelling along the proximal articulation with the radius surface (PRU). The reactive bone has a ‘cauliflower-like’ appearance. The olecranon process has been highly degraded by the pathological condition. Large lesions can be seen on the proximal surface (red circle). The red box marks the area scanned using XMT in figure 2.

**Figure 2.**
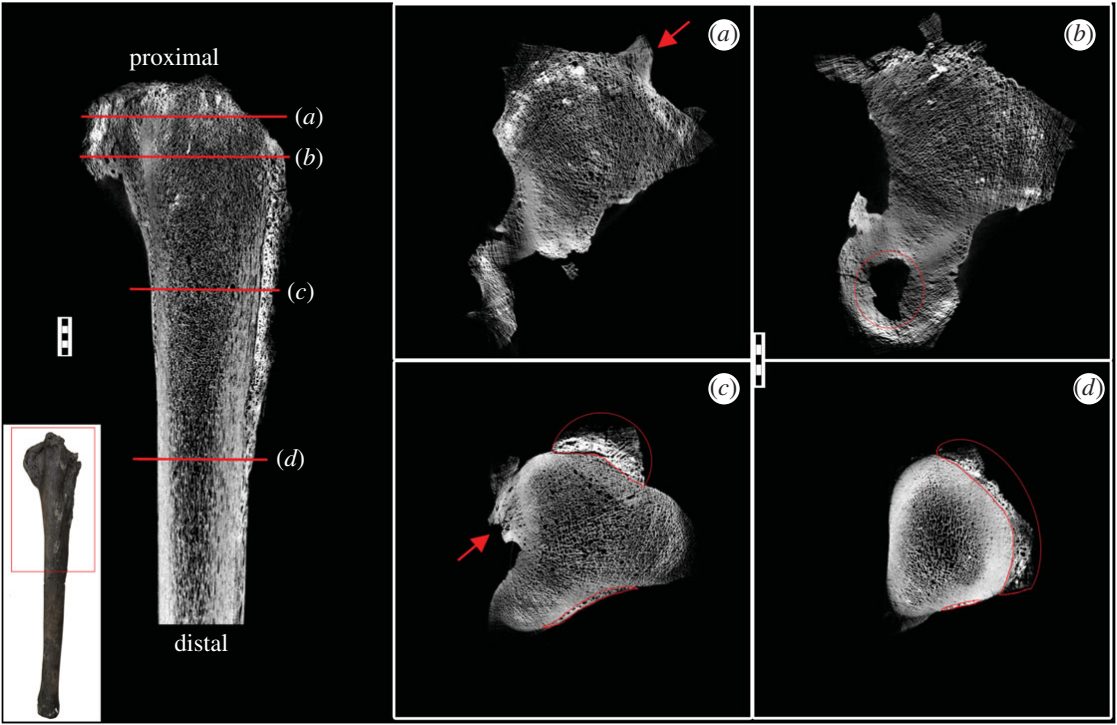
XMT scans of the NJSM GP11961 ulna in longitudinal and transverse (*a*–*d*) views. The area scanned is indicated on the gross picture insert. Locations for the transverse sections are marked by red lines on the longitudinal section. Well-developed enthesiophytes are seen in two different locations (*a,c*; red arrows). Necrosis is seen along the proximal articulation surface (*b*; circled in red). Reactive bone growth is present throughout the first proximal half of the element, tapering out distally (lateral, *c,d* circled in red). Scale bar, 10 mm.

The affected area is most severe for the first approximately 14 cm of the proximal end, but extends almost half the total length of the element (approx. 31 cm; figure 1). There is heavy remodelling resulting in a cauliflower-like texture present in some areas, especially around the articulation surfaces (figure 1). A large lesion and several smaller lesions evident of necrosis are seen on the proximal articulation surface (figure 1; red circle). XMT scans reveal the extent of the lesions especially within the expansion along the radius/ulna articular surface (figure 2*b*). Along the olecranon process, the cortical wall is non-existent except for a small patch on the radial articulation surface. Internal examination also revealed that the pathology consists of reactive bone growth (figure 2). A large enthesophyte (abnormal bony projection along tendon/ligament attachments [6]) is located on the outer surface (figure 2*a*). Radial bone growth is seen along the entire medial surface of the ulna, spreading distally to cover the circumference of the ulna (figure 2). The radial growth then decreases distally, tapering completely away to a small area on the radial articulation surface, where there are two large protrusions on either side of the lateral process (figure 2*c,d*). One of these protrusions consists of unorganized, random tissue orientation (figure 2*c*, circled red). The other is in a more compact, unidirectional orientation, suggesting a second enthesophyte (figure 2*c*, red arrow).

### 3.2. Radius

**Figure 3.**
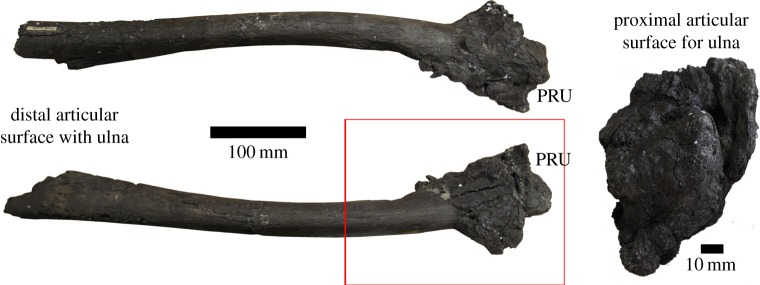
Pathological radius from a hadrosaur (NJSM GP11961) showing heavy reactive bone growth on the proximal articulation surfaces. The proximal articulation surface with the ulna (PRU) shows extensive pathological bone growth, giving the element a ‘cauliflower-like’ appearance. The distal articular surface with the ulna has mostly eroded away due to taphonomic effects. The red box marks the area scanned using XMT in figure 4.

**Figure 4.**
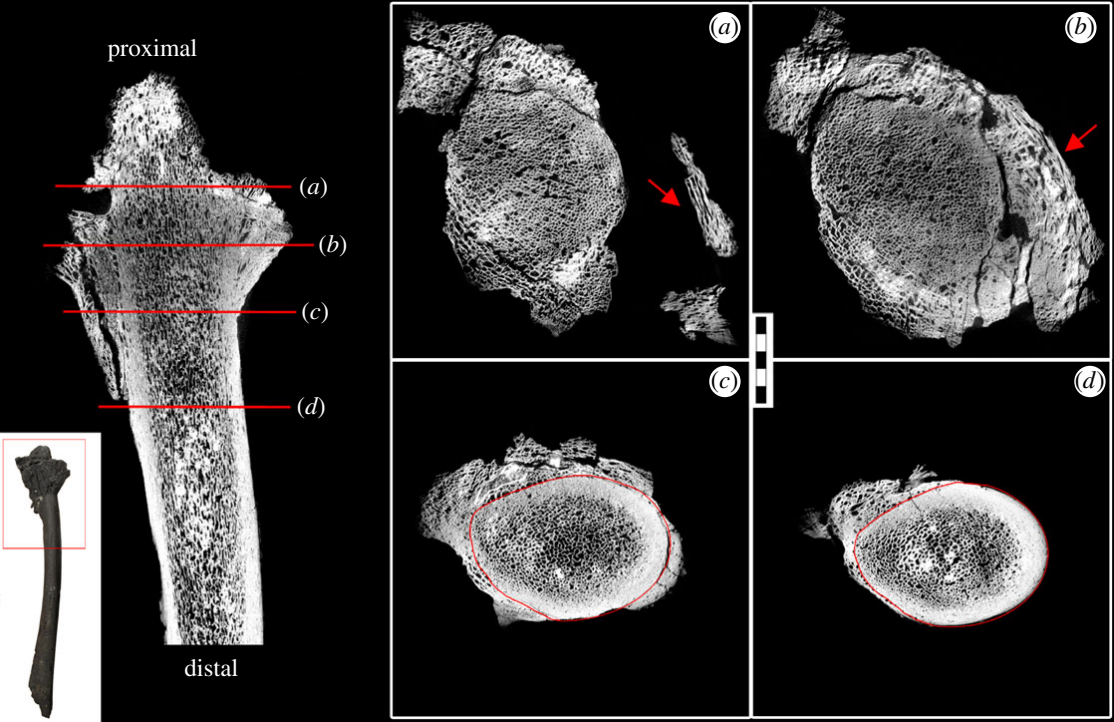
XMT scans of the NJSM GP11961 radius in longitudinal and transverse (*a*–*d*) views. The area scanned is indicated on the gross picture insert. Locations for the transverse sections are marked by red lines on the longitudinal section. The original outline of the bone is completely unrecognizable on the proximal end, with severe, radial bone growth (*a,b*; red arrow). There is a large gap between the pathological growth and the original cortex of the radius (*a,b*; red arrows). The outline of the radius becomes clear with the appearance of the normal cortical bone structure moving distally (*c,d*; original radius outline circled in red). Scale bar, 10 mm.

The pathological condition of the radius is more localized than in the ulna. Unlike the ulna, the pathological bone growth is easily observable, even in gross examination, and has a sharp contact with the normal bone, creating an easily discernable outline (figure 3). XMT revealed that despite the clear distinction on the outside, the internal structure of the radius is a mess of ‘frothy’ bone with the outline of the radius unclear for the first proximal 3 cm (one-third) of the pathology (figure 4*a*,*b*). Extensive pathological growth is present distally and is composed of two morphologically distinct types of tissue. One is very porous; while the other has a wood-like appearance with long struts of bone that have grains following the circumference of the radius (figure 4*a*,*b*, red arrow). There is a large gap between the normal cortical wall of the radius and these ‘woody’ growths, with the possible origin point for these growths located much more distally. Eventually, the typical dense cortical bone associated with the normal radius cortex dominates and pathological growth decreases, which persists up to 20 cm from the proximal end of the radius (figure 4*c*,*d*).

## 4. Diagnosis

The ulna and radius of NJSM GP11961 show signs of both excessive bone necrosis and reactive (pathological) bone growth, which can be attributed to several conditions seen in reptiles and birds (table 1). Neoplasia can be excluded since it is both rare (in wild individuals) and is a reactive (pathological growth) condition [14–16,19,30]. Osteopetrosis is another exclusively reactive condition and is only found in the diaphysis of long bones in poultry [20]. Thus, both the location of the pathology (diaphysis versus joints) and its occurrence only in poultry make it an unlikely cause. Gout, a common condition in reptiles, causes lytic lesions within the joints through the deposition of urate crystals [19,21]. However, gout is strictly destructive and not associated with reactive bone growth. Tuberculosis can also be ruled out since it is also a strictly destructive condition [16,29].

The most likely explanation for the given pathology is a form of osteoarthritis, which is defined as any condition affecting movable joints by deterioration of articular cartilage, osteophyte (bone spur) formation, bone remodelling and changes in the surrounding joint tissue [31]. Such conditions are usually localized in reptiles, unlike in humans where these conditions can spread throughout the body [26]. Osteoarthritis in avians and reptiles is associated with additional complications such as disease, trauma or infection [16,19,32]. Two osteoarthritic conditions associated with necrosis and reactive bone growth are osteomyelitis and septic arthritis. The main difference between the two is that osteomyelitis is usually associated with the infection of ossified tissue (e.g. bone), whereas septic arthritis is usually associated with the infection of non-ossified tissue (e.g. cartilage) [6]. Septic arthritis does affect bone tissue as it can result in bone erosion and ankylosing due to the destruction of articular cartilage [6]. However, this distinction is not always made clear in veterinary cases where both conditions are often labelled as osteomyelitis (e.g. [15,22]).

In the case of the NJSM GP11961, the erosional attributes, combined with the highly reactive bone growth and the fusion of the elements suggest that this condition is septic arthritis. Our interpretation is necrosis and remodelling of the elbow joint was caused by the loss of articular cartilage due to septic arthritis. The weakening and eventual destruction of the joint caused pathologic bone growth and ankylosis of bone acting in response to the pathology in order to strengthen the joint. Destruction of the olecranon process also resulted in the formation of the two enthesophytes located along the ulna for alternative tendon and ligament attachment sites [6].

Septic arthritis has not been previously noted in dinosaurs, though osteomyelitis has been identified in all dinosaurian groups including other hadrosauroids [4,33]. In this study, osteomyelitis was excluded due to the location around the elbow joint and reactive nature of the condition since, unlike in mammals, there is little to no periosteal reactive bone growth associated with osteomyelitis in birds and reptiles [15]. On the other hand, septic arthritis is reactive in reptiles and can lead to ankylosis as has been previously noted [28]. Although there are reports of highly reactive osteomyelitis in birds [23], the most reactive forms are chronic and fungal arthritis, which may have been initially caused by septic arthritis [15]. Thus, we interpret septic arthritis as the cause of the pathology found in NJSM GP11961, making this the first documented case of this condition within the non-avian Dinosauria. This interpretation was only made possible by using the combination of internal morphologies achieved through XMT, as well as the use of comparable extant species for interpretation of the condition.

## 5. Conclusion

Palaeopathological studies allow unique insights into extinct animal physiology through the comparative study of conditions and modes of immunity seen in comparable extant species. The application of non-destructive internal examination through the use of high-resolution XMT scans are crucial for such studies and allow for the sampling and study of many more specimens and species. In this study, XMT scans allowed for the detailed diagnosis of a rare and fragile hadrosaur specimen from New Jersey. We diagnose the palaeopathology as septic arthritis; the first case documented in the Dinosauria. This diagnosis was only possible due to the internal examination of the pathology by XMT, which has also ensured that such information will not be lost thanks to the digital data now being accessible.

## Data accessibility

All scan data are available through Dryad at: http://dx.doi.org/10.5061/dryad.b84nh.
